# Supplementary material for: Mechanical force of uterine occupation enables large vesicle extrusion from proteostressed maternal neurons
Source: eLife. 2024 Sep 10;13:RP95443. doi: 10.7554/eLife.95443 (PMC11386954; doi:10.7554/eLife.95443)
Supplement: Figure 1—source data 1. [file elife-95443-fig1-data1.docx]

Figure 1-source data:

Panel B

| Adult Day | Exopher: % | | | | | | | | |
| --- | --- | --- | --- | --- | --- | --- | --- | --- | --- |
| 1 | 0 | 0 | 2 | 28 | 8 | 6 | 4 | 4 | 2 |
| 2 | 20 | 26 | 34 | 18 | 20 | 8 | 16 | 8 | 6 |
| 3 | 40 | 18 | 14 | 8 | 14 | 10 | 6 | 12 | 8 |
| 4 | 6 | 4 | 12 | 10 | 12 | 4 | 8 | 4 | 4 |
| 5 | 4 | 8 | 6 | 8 | 8 | 6 | 4 | 2 | 10 |
| Sample size: | 50 worms for each data point. | | | | | | | | |

|  | Progeny: count per hermaphrodite | | | | | | | | | |
| --- | --- | --- | --- | --- | --- | --- | --- | --- | --- | --- |
| Adult day | worm1 | worm2 | worm3 | worm4 | worm5 | worm6 | worm7 | worm8 | worm9 | worm  10 |
| 1 | 37 | 31 | 43 | 46 | 33 | 44 | 39 | 44 | 23 | 38 |
| 2 | 169 | 172 | 170 | 177 | 170 | 186 | 174 | 167 | 166 | 80 |
| 3 | 62 | 103 | 103 | 94 | 89 | 113 | 109 | 90 | 78 | 90 |
| 4 | 11 | 16 | 5 | 13 | 16 | 7 | 20 | 5 | 3 | 82 |
| 5 | 23 | 5 | 1 | 1 | 3 | 13 | 2 | 7 | 1 | 18 |

Panel C:

|  |  |  |  | Sample size | | Cochran–Mantel–Haenszel test |
| --- | --- | --- | --- | --- | --- | --- |
| Exopher: % | Control | FUdR |  | Control | FUdR |  |
| Trial 1 | 18.1818182 | 4.54545455 |  | 55 | 42 |  |
| Trial 2 | 16.1290323 | 0 |  | 62 | 38 |  |
| Trial 3 | 12.5 | 0 |  | 64 | 40 | *p* = 0.00015 |

Panel E:

|  | Exopher: % | | | | | |
| --- | --- | --- | --- | --- | --- | --- |
| Adult Day | wild type | | | *glp-4(ts)* | | |
| 1 | 4 | 4 | 2 | 0 | 0 | 4 |
| 2 | 16 | 8 | 6 | 0 | 0 | 0 |
| 3 | 6 | 12 | 8 | 4 | 4 | 4 |
| 4 | 8 | 4 | 4 | 0 | 2 | 0 |
| 5 | 4 | 2 | 10 | 2 | 0 | 0 |
| Sample size | | 50 worms at each data point | | | | |
| Cochran–Mantel–Haenszel test | | | | | | |
| Adult day 1 | *p* = 0.45 |  |  |  |  |  |
| Adult day 2 | *p* = 0.002 |  |  |  |  |  |
| Adult day 3 | *p* = 0.16 |  |  |  |  |  |
| Adult day 4 | *p* = 0.04 |  |  |  |  |  |
| Adult day 5 | *p* = 0.04 |  |  |  |  |  |

Panel F:

|  | Exopher: % | | | | | |
| --- | --- | --- | --- | --- | --- | --- |
| Adult Day | wild type | | | *fem-3(gf)* | | |
| 1 | 0 | 0 | 0 | 0 | 0 | 0 |
| 2 | 8 | 12.24 | 11.6 | 0 | 0 | 0 |
| 3 | 5 | 8.3 | 7.8 | 0 | 0 | 0 |
| 4 | 5 | 4.25 | 5.9 | 0 | 2 | 0 |
| 5 | 4 | 4 | 0 | 0 | 0 | 0 |
|  |  |  |  |  |  |  |
|  | Sample size | | | | | |
| Adult Day | wild type | | | *fem-3(gf)* | | |
| 1 | 50 | 50 | 50 | 50 | 50 | 50 |
| 2 | 50 | 49 | 48 | 50 | 50 | 50 |
| 3 | 40 | 48 | 51 | 50 | 50 | 50 |
| 4 | 80 | 47 | 51 | 50 | 50 | 50 |
| 5 | 50 | 50 | 50 | 50 | 50 | 50 |
|  |  |  |  |  |  |  |
| Adult Day | Cochran–Mantel–Haenszel test | | | | | |
| 1 | N.A. | | | | | |
| 2 | *p* = 0.000186 | | | | | |
| 3 | *p* = 0.0029 | | | | | |
| 4 | *p* = 0.047 | | | | | |
| 5 | *p* = 0.13 | | | | | |

Panel G:

|  | Exopher: % | | | | | |
| --- | --- | --- | --- | --- | --- | --- |
| Adult day | wild type | | | *fem-1(lf)* | | |
| 1 | 16 | 12 | 8 | 0 | 0 | 0 |
| 2 | 12 | 16 | 2 | 0 | 0 | 0 |
|  |  |  |  |  |  |  |
| Sample size | 50 worms at each data point | | | | | |
| Adult day | Cochran–Mantel–Haenszel test | | | | | |
| 1 | *p* = 0.00004 | | | | | |
| 2 | *p* = 0.000196 | | | | | |

Panel H:

| Adult Day | control | | | | | | non-differentiated sperm | | | | | |
| --- | --- | --- | --- | --- | --- | --- | --- | --- | --- | --- | --- | --- |
| 1 | 4 | 6 | 2 | 0 |  |  | 0 | 0 | 0 | 0 |  |  |
| 2 | 40 | 34 | 38 | 20 | 20 | 6 | 0 | 0 | 0 | 0 | 0 | 0 |
| 3 | 22 | 16 | 24 | 4 | 16 | 8 | 0 | 0 | 0 | 0 | 0 | 0 |
| 4 | 8 | 6 | 10 | 6 | 0 | 2 | 0 | 0 | 0 | 0 | 0 | 0 |
| 5 | 4 | 6 | 2 | 6 | 2 | 2 | 0 | 0 | 0 | 0 | 0 | 0 |
|  |  |  |  |  |  |  |  |  |  |  |  |  |
| Sample size | 50 worms at each data point | | | | | | | | | | | |
|  | Cochran–Mantel–Haenszel test | | | | | | | | | | | |
| Adult Day |  |  |  |  |  |  |  |  |  |  |  |  |
| 1 | *p* = 0.013 | | | | | | | | | | | |
| 2 | *p* < 0.0001 | | | | | | | | | | | |
| 3 | *p* < 0.0001 | | | | | | | | | | | |
| 4 | *p* = 0.00014 | | | | | | | | | | | |
| 5 | *p* = 0.000001 | | | | | | | | | | | |
