# Supplementary material for: Mechanical force of uterine occupation enables large vesicle extrusion from proteostressed maternal neurons
Source: eLife. 2024 Sep 10;13:RP95443. doi: 10.7554/eLife.95443 (PMC11386954; doi:10.7554/eLife.95443)
Supplement: Figure 2—source data 1. [file elife-95443-fig2-data1.docx]

Figure 2-source data

B.

|  | Exopher: % | |
| --- | --- | --- |
|  | Control | \| *cbd-1* RNAi \| \| --- \| |
| Trial 1 | 6 | 0 |
| Trial 2 | 6 | 0 |
| Trial 3 | 10 | 0 |
|  |  |  |
| Sample size | 50 | 50 |
| **Cochran–Mantel–Haenszel test** | | |
| *p* = 0.002 | | |

C.

|  | Exopher: % | |
| --- | --- | --- |
|  | Control | \| *gna-2* RNAi \| \| --- \| |
| Trial 1 | 10 | 0 |
| Trial 2 | 6 | 0 |
| Trial 3 | 8 | 0 |
|  |  |  |
| Sample size | 50 | 50 |
| **Cochran–Mantel–Haenszel test** |  |  |
| *p* = 0.0012 |  |  |

D.

|  | Exopher: % | |
| --- | --- | --- |
|  | Wild type | \| *gna-2(∆)* \| \| --- \| |
| Trial 1 | 28 | 0 |
| Trial 2 | 6 | 0 |
| Trial 3 | 24 | 0 |
|  |  |  |
| Sample size | 50 | 50 |
| **Cochran–Mantel–Haenszel test** |  |  |
| *p* < 0.0001 |  |  |

E.

|  | Exopher: % | |
| --- | --- | --- |
|  | Control | \| *perm-1* RNAi \| \| --- \| |
| Trial 1 | 6 | 0 |
| Trial 2 | 6 | 2 |
| Trial 3 | 10 | 2 |
|  |  |  |
| Sample size | 50 | 50 |
| **Cochran–Mantel–Haenszel test** |  |  |
| *p* = 0.024 |  |  |

G.

|  | Exopher: % | | | | | | | | **Cochran–Mantel–Haenszel test** |
| --- | --- | --- | --- | --- | --- | --- | --- | --- | --- |
|  | Control (EV) | | | | RNAi | | | |  |
| *pfn-1* | 10 | 6 | 8 |  | 0 | 4 | 6 |  | *p* = 0.013 |
| *pod-1* | 6 | 22 | 10 |  | 0 | 0 | 0 |  | *p* < 0.0001 |

H.

|  | Exopher: % | | | | | | **Cochran–Mantel–Haenszel test** |
| --- | --- | --- | --- | --- | --- | --- | --- |
|  | Control (EV) | | | RNAi | | |  |
| *mex-3* | 6 | 22 | 10 | 8 | 6 | 6 | *p* = 0.04 |
| *mom-2* | 6 | 22 | 10 | 9 | 15 | 16 | *p* = 0.87 |
| *end-1/-3* | 6 | 22 | 10 | 8 | 8 | 16 | *p* = 0.39 |
| *gad-1* | 12 | 12 | 6 | 10 | 8 | 10 | *p* = 0.85 |
