# Supplementary material for: Mechanical force of uterine occupation enables large vesicle extrusion from proteostressed maternal neurons
Source: eLife. 2024 Sep 10;13:RP95443. doi: 10.7554/eLife.95443 (PMC11386954; doi:10.7554/eLife.95443)
Supplement: Figure 3—source data 1. [file elife-95443-fig3-data1.docx]

Figure 3-source data

A.

| Adult day | control (no male) | | | | | | | | | | | |
| --- | --- | --- | --- | --- | --- | --- | --- | --- | --- | --- | --- | --- |
| 1 | 53 | 47 | 38 | 37 | 33 | 42 | 43 | 45 | 38 | 37 | 44 | 46 |
| 2 | 137 | 147 | 160 | 109 | 72 | 187 | 148 | 160 | 161 | 162 | 145 | 160 |
| 3 | 13 | 65 | 55 | 29 | 26 | 42 | 14 | 75 | 55 | 91 | 41 | 54 |
| 4 | 12 | 19 | 3 | 9 | 17 | 12 | 5 | 4 | 3 | 6 | 1 | 8 |
| 5 | 16 | 15 | 1 | 5 | 9 | 8 | 16 | 0 | 0 | 2 | 2 | 10 |
| 6 | 9 | 2 | 3 | 6 | 3 | 7 | 7 | 0 | 6 | 4 | 0 | 1 |
|  |  |  |  |  |  |  |  |  |  |  |  |  |
|  | + males (fertile) | | | | | | | | | | | |
| 1 | 54 | 29 | 45 | 47 | 52 | 51 | 36 | 34 | 41 | 37 | 48 | 43 |
| 2 | 174 | 128 | 160 | 152 | 110 | 163 | 151 | 132 | 130 | 150 | 172 | 162 |
| 3 | 58 | 60 | 66 | 22 | 43 | 61 | 33 | 41 | 38 | 47 | 43 | 38 |
| 4 | 2 | 6 | 6 | 10 | 6 | 4 | 3 | 23 | 8 | 10 | 24 | 0 |
| 5 | 36 | 53 | 39 | 31 | 3 | 12 | 61 | 73 | 62 | 38 | 3 | 68 |
| 6 | 28 | 30 | 37 | 40 | 0 | 44 | 48 | 69 | 22 | 31 | 0 | 41 |
|  |  |  |  |  |  |  |  |  |  |  |  |  |
| **Two-way ANOVA** | | | | | | | | | | | | |
| Adult day 4: control vs. + males | | | | | | | *p* < 0.0001 | | | | | |
| Adult day 5: control vs. + males | | | | | | | *p* < 0.0001 | | | | | |

B.

|  | Exopher: % | | | | | | | | |
| --- | --- | --- | --- | --- | --- | --- | --- | --- | --- |
| Adult day | control (no male) | | | + male (sterile) | | | + male (fertile) | | |
| 1 | 0 | 2 | 4 |  |  |  |  |  |  |
| 2 | 19 | 28 | 28 |  |  |  |  |  |  |
| 3 | 30 | 24 | 20 | 30 | 24 | 20 | 30 | 24 | 20 |
| 4 | 18 | 0 | 14 | 16 | 10 | 12 | 16 | 20 | 24 |
| 5 | 0 | 6 | 12 | 2 | 6 | 10 | 26 | 32 | 44 |
| 6 | 0 | 0 | 6 | 4 | 8 | 6 | 18 | 44 | 8 |
|  |  |  |  |  |  |  |  |  |  |
| **Cochran–Mantel–Haenszel test** | | | | | | | | | |
| Adult day 5: + male (fertile) vs. the other two groups | | | | | | | *p* < 0.0001 | | |

D.

|  | Exopher: % | |
| --- | --- | --- |
| Trial# | wild type | *egl-9(Δ)* |
| 1 | 14 | 60 |
| 2 | 18 | 54 |
| 3 | 30 | 46 |
|  |  |  |
| **Cochran–Mantel–Haenszel test** | | |
| *p* < 0.0001 | | |

E.

|  | Exopher: % | |
| --- | --- | --- |
| Trial# | wild type | *egl-3(Δ)* |
| 1 | 22 | 84 |
| 2 | 16 | 82 |
| 3 | 20 | 84 |
| **Cochran–Mantel–Haenszel test** | | |
| *p* < 0.0001 | | |

F.

|  | Exopher: % | |
| --- | --- | --- |
| Trial# | wild type | *sem-2(rf)* |
| 1 | 4 | 80 |
| 2 | 12 | 84 |
| 3 | 2 | 84 |
| **Cochran–Mantel–Haenszel test** | |  |
| *p* < 0.0001 | |  |

G.

|  | Exopher: % | |
| --- | --- | --- |
| Trial# | control | *lin-39* RNAi |
| 1 | 0 | 76 |
| 2 | 16 | 74 |
| 3 | 10 | 72 |
| 4 | 12 | 40 |
| 5 | 2 | 48 |
| 6 | 0 | 60 |
| **Cochran–Mantel–Haenszel test** | | |
| *p* < 0.0001 | | |

H.

|  | Exopher: % | |
| --- | --- | --- |
| Trial# | control | *lin-39* RNAi |
| 1 | 0 | 74 |
| 2 | 0 | 52 |
| 3 | 0 | 58 |
| **Cochran–Mantel–Haenszel test** | | |
| *p* < 0.0001 | | |

I.

|  | Exopher: % | | | |
| --- | --- | --- | --- | --- |
| Trial# | M9 | 20 mM OA | M9 | 4 mM  5-HT |
| 1 | 4.761904762 | 31.42857143 | 34.92063492 | 10.71428571 |
| 2 | 14.03508772 | 30.50847458 | 40 | 6.25 |
| 3 | 13.95348837 | 31.25 | 53.7037037 | 29.50819672 |
|  |  |  |  |  |
| **Cochran–Mantel–Haenszel test** | | | | |
|  | *p* < 0.001 | | *p* < 0.0001 | |

J.

|  | Exopher: % | |
| --- | --- | --- |
| Trial# | wild type | *goa-1(Δ)* |
| 1 | 14 | 4 |
| 2 | 8 | 2 |
| 3 | 10 | 2 |
|  |  |  |
| **Cochran–Mantel–Haenszel test** | | |
| *p* = 0.01 | | |
