# Supplementary material for: Mechanical force of uterine occupation enables large vesicle extrusion from proteostressed maternal neurons
Source: eLife. 2024 Sep 10;13:RP95443. doi: 10.7554/eLife.95443 (PMC11386954; doi:10.7554/eLife.95443)
Supplement: Figure 4—source data 1. [file elife-95443-fig4-data1.docx]

Figure 4-source data:

D.

|  | ALMR within Egg Zone | | | ALMR outside of Egg Zone | | |
| --- | --- | --- | --- | --- | --- | --- |
| % | Mean | SD | N | Mean | SD | N |
| No Exopher | 70.33 |  | 64 | 29.67 |  | 27 |
| Exopher + | 94.59 |  | 35 | 5.41 |  | 2 |
|  |  |  |  |  |  |  |
| **Chi-Square test** | | | | | *p* < 0.01 | |

F.

|  | Exopher: % | |
| --- | --- | --- |
| Trial# | Anterior to AVM | Posterior to AVM |
| 1 | 2.941176 | 85.185185 |
| 2 | 15.789474 | 85 |
| 3 | 0 | 51.515152 |
|  |  |  |
| **Cochran–Mantel–Haenszel test** | | |
|  | *p* < 0.0001 | |

G.

|  | Exopher: % | | | | | | | |
| --- | --- | --- | --- | --- | --- | --- | --- | --- |
|  | wild type | | | | *sem-2(rf)* | | | |
| AVM | 0 | 0 | 0 | 0 | 48 | 70 | 46 | 64 |
| ALMR | 2 | 2 | 2 | 4 | 94 | 92 | 82 | 96 |
| ALML | 0 | 0 | 2 | 0 | 44 | 42 | 28 | 34 |
| PVM | 0 | 0 | 0 | 0 | 58 | 48 | 30 | 50 |
| PLMs | 0 | 0 | 0 | 0 | 0 | 0 | 0 | 0 |
|  |  |  |  |  |  |  |  |  |
| Sample size  (per group in each trail) | 50 | 50 | 50 | 50 | 50 | 50 | 50 | 50 |
