# Supplementary material for: Mechanical force of uterine occupation enables large vesicle extrusion from proteostressed maternal neurons
Source: eLife. 2024 Sep 10;13:RP95443. doi: 10.7554/eLife.95443 (PMC11386954; doi:10.7554/eLife.95443)
Supplement: Figure 5—source data 1. [file elife-95443-fig5-data1.docx]

Figure 5-source data:

B.

| Length: um | |
| --- | --- |
| wild type | *egl-3(Δ)* |
| 381.161 | 526.999 |
| 419.272 | 455.597 |
| 320.65 | 375.239 |
| 293.58 | 628.338 |
| 436.033 | 359.149 |
| 269.389 | 406.185 |
| 333.057 | 456.33 |
| 397.83 | 559.362 |
| 419.842 | 403.809 |
| 353.917 | 457.169 |
| 290.822 | 397.354 |
| 389.531 | 386.167 |
| 412.879 | 402.561 |
| 222.703 | 356.608 |
| 362.748 | 606.857 |
| 282.973 | 416.173 |
| 333.735 | 386.211 |
|  | 474.62 |
|  | 425.049 |
|  | 392.115 |
|  |  |
| *t-*test | *p =* 0.0003 |

C.

| Length: um | |
| --- | --- |
| wild type | *egl-9(Δ)* |
| 314.458 | 423.937 |
| 429.696 | 441.279 |
| 406.241 | 413.426 |
| 394.272 | 560.367 |
| 346.108 | 372.309 |
| 427.58 | 403.153 |
| 374.741 | 561.16 |
| 423.403 | 594.016 |
| 465.257 | 482.859 |
| 444.265 | 521.865 |
| 325.079 | 686.196 |
| 383.318 | 471.575 |
| 362.105 | 476.772 |
| 386.528 | 600.41 |
| 404.447 | 454.428 |
|  | 571.625 |
|  | 434.043 |
|  | 406.582 |
|  | 528.27 |
|  | 548.994 |
|  |  |
| *t-*test | *p* < 0.0001 |

D.

| Length: um | |
| --- | --- |
| wild type | *sem-2(rf)* |
| 278.939 | 455.729 |
| 210.56 | 606.019 |
| 182.957 | 438.468 |
| 340.533 | 379.522 |
| 226.766 | 507.204 |
| 337.59 | 382.939 |
| 313.48 | 575.973 |
| 293.332 | 514.767 |
| 308.193 | 546.871 |
| 411.434 | 532.227 |
| 347.468 | 417.181 |
| 276.581 | 576.535 |
| 325.758 | 553.978 |
| 355.382 | 605.253 |
| 221.435 | 648.696 |
|  | 580.753 |
|  |  |
| *t-*test | *p* < 0.0001 |

E.

| Length: um | |
| --- | --- |
| EV control | *cbd-1* RNAi |
| 543.557 | 203.94 |
| 317.704 | 158.845 |
| 326.475 | 146.462 |
| 517.338 | 210.677 |
| 424.38 | 123.557 |
| 325.444 | 214.615 |
| 418.569 | 203.061 |
| 362.498 | 139.607 |
| 393.923 | 212.579 |
| 437.172 | 174.455 |
| 395.19 | 154.61 |
| 361.294 | 111.495 |
| 306.131 | 244.43 |
| 348.144 | 161.167 |
| 402.366 | 168.292 |
|  | 223.126 |
|  | 170.661 |
|  |  |
| *t-*test | *p* < 0.0001 |

F.

| Length: um | |
| --- | --- |
| wild type | wild type  (no sperm) |
| 397.759 | 244.224 |
| 351.579 | 275.226 |
| 468.022 | 188.024 |
| 419.853 | 200.369 |
| 423.264 | 197.809 |
| 305.32 | 158.949 |
| 439.164 | 208.627 |
| 475.432 | 209.984 |
| 372.054 | 209.928 |
| 420.789 | 108.89 |
| 389.551 | 228.388 |
| 377.082 | 160.042 |
| 345.884 | 179.834 |
| 405.051 | 257.022 |
| 399.404 |  |
| 389.632 |  |
|  |  |
| *t-*test | *p* < 0.0001 |

G.

| Length: um | |
| --- | --- |
| wild type | *goa-1(Δ)* |
| 300.925867 | 226.173867 |
| 276.027733 | 174.721067 |
| 273.181867 | 199.111467 |
| 269.163733 | 197.0272 |
| 258.416 | 142.5856 |
| 314.525867 | 151.7792 |
| 280.493867 | 199.304533 |
| 267.176533 | 207.837867 |
| 273.912533 | 187.870933 |
| 255.010133 | 217.8208 |
| 249.7728 | 197.543467 |
| 266.8832 | 151.018667 |
| 252.1696 | 146.426667 |
| 280.517333 | 152.718933 |
| 266.0288 | 171.2288 |
| 255.2384 | 195.674667 |
| 306.5056 | 171.396267 |
| 278.416 | 175.792 |
| 220.593067 | 153.335467 |
| 284.813867 | 160.443733 |
|  |  |
| *t-*test | *p* < 0.0001 |

H.

| Length: um | | | |
| --- | --- | --- | --- |
| EV control | *mex-3* RNAi | EV control | *gad-1* RNAi |
| 414.796 | 324.496 | 345.38 | 385.181 |
| 366.162 | 470.849 | 430.479 | 351.559 |
| 364.846 | 327.069 | 431.252 | 372.376 |
| 301.066 | 351.944 | 409.303 | 394.809 |
| 369.282 | 300.924 | 386.223 | 351.798 |
| 219.648 | 338.131 | 367.249 | 394.831 |
| 440.114 | 393.574 | 352.328 | 454.756 |
| 370.092 | 399.5 | 365.114 | 438.607 |
| 381.699 | 457.243 | 313.658 | 372.266 |
| 382.588 | 481.509 | 354.984 | 376.67 |
| 323.333 | 352.772 | 377.554 | 352.886 |
| 488.178 | 330.476 | 547.253 | 413.099 |
| 450.899 | 530.698 | 332.786 | 256.142 |
| 392.147 | 355.375 | 374.591 | 319.721 |
| 409.639 | 388.038 | 382.971 | 351.396 |
| 292.864 | 310.056 | 429.067 | 350.433 |
| 303.545 | 331.917 | 344.854 | 348.914 |
| 301.372 | 293.26 |  |  |
| 384.692 | 381.086 |  |  |
| 447.669 | 421.65 |  |  |
|  |  |  |  |
| **One way ANOVA** | | | |
| *p* = 0.9833 vs. EV control | | *p* = 0.8740 vs. EV control | |
