# Supplementary material for: Mechanical force of uterine occupation enables large vesicle extrusion from proteostressed maternal neurons
Source: eLife. 2024 Sep 10;13:RP95443. doi: 10.7554/eLife.95443 (PMC11386954; doi:10.7554/eLife.95443)
Supplement: Figure 6—source data 1. [file elife-95443-fig6-data1.docx]

Figure 6-source data: **Numerical data**

A.

| Exopher: % | | | | | | |
| --- | --- | --- | --- | --- | --- | --- |
|  | WT@day 2 | | | *sem-2(lf)*@day 1 | | |
| WT eggs | 6 | 12 | 10 | 72 | 92 | 80 |
| *cbd-1(*RNAi) | 0 | 0 | 2 | 56 | 32 | 50 |
|  |  |  |  |  |  |  |
| Sample size  (per group) | 50 | 50 | 50 | 50 | 50 | 50 |
| **Cochran–Mantel–Haenszel** (**CMH**) **test** | | | | | | |
| WT@day 2 vs. *sem-2(lf)*@day 1 for WT eggs | | | | | *p* < 0.0001 | |
| WT@day 2 vs. *sem-2(lf)*@day 1 for *cbd-1(RNAi)* | | | | | *p* < 0.0001 | |

B.

| Length: um | |
| --- | --- |
| *cbd-1(RNAi)* | |
| wild type | *sem-2(rf)* |
| 203.94 | 650.874 |
| 158.845 | 533.601 |
| 146.462 | 603.889 |
| 210.677 | 656.12 |
| 123.557 | 590.319 |
| 214.615 | 701.667 |
| 203.061 | 648.959 |
| 139.607 | 563.628 |
| 212.579 | 656.337 |
| 174.455 | 614.611 |
| 154.61 | 572.64 |
| 111.495 | 517.209 |
| 244.43 | 653.267 |
| 161.167 | 629.588 |
| 168.292 | 679.319 |
| 223.126 | 701.787 |
| 170.661 | 582.586 |
|  | 618.729 |
|  | 515.255 |
|  | 545.332 |
|  |  |
| ***t*-test** | *p* < 0.0001 |

C.

| Exopher: % | | | | | | | | | |
| --- | --- | --- | --- | --- | --- | --- | --- | --- | --- |
|  | L4 | | | L4 + 24hr | | | L4 + 48hr | | |
| normal sperm | 0 | 0 | 0 | 92 | 94 | 92 |  |  |  |
| no sperm | 0 | 0 | 0 | 32 | 70 | 46 | 82 | 80 | 96 |
|  |  |  |  |  |  |  |  |  |  |
| Sample size  (per group) | 50 | 50 | 50 | 50 | 50 | 50 | 50 | 50 | 50 |

D.

| Length: um | |
| --- | --- |
| wild type | *sem-2(rf)* |
| 244.224 | 520.291 |
| 275.226 | 554.331 |
| 188.024 | 708.796 |
| 200.369 | 645.746 |
| 197.809 | 585.414 |
| 158.949 | 598.682 |
| 208.627 | 615.826 |
| 209.984 | 632.794 |
| 209.928 | 394.893 |
| 108.89 | 659.33 |
| 228.388 | 788.638 |
| 160.042 | 560.79 |
| 179.834 | 476.547 |
| 257.022 | 576.156 |
|  | 414.902 |
|  |  |
| ***t*-test** | *p* < 0.0001 |

E.

| Exopher: % | | | | | | |
| --- | --- | --- | --- | --- | --- | --- |
|  | EV control | | | *lin-39* RNAi | | |
| Adult day 1 | 0 | 0 | 0 | 10 | 26 | 8 |
| Adult day 2 | 0 | 0 | 0 | 50 | 48 | 52 |
|  |  |  |  |  |  |  |
| Sample size  (per group) | 50 | 50 | 50 | 50 | 50 | 50 |
| **Cochran–Mantel–Haenszel** (**CMH**) **test** | | | | | | |
| Adult day 1 | *p* < 0.0001 | | | | | |
| Adult day 2 | *p* < 0.0001 | | | | | |

F.

| Length: um | |
| --- | --- |
| EV control | *lin-39* RNAi |
| 146.194 | 555.466 |
| 178.625 | 563.127 |
| 192.09 | 662.817 |
| 143.052 | 527.639 |
| 181.085 | 454.082 |
| 203.542 | 563.879 |
| 145.91 | 587.093 |
| 272.845 | 510.248 |
| 194.011 | 726.605 |
| 249.754 | 525.57 |
| 160.065 | 531.404 |
| 134.502 | 694.139 |
| 157.529 | 392.752 |
| 194.774 | 514.044 |
| 220.099 | 574.554 |
| 198.178 | 441.975 |
|  | 537.705 |
|  |  |
| ***t*-test** | *p* < 0.0001 |

G.

| Exopher: % | | | | | | |
| --- | --- | --- | --- | --- | --- | --- |
|  | EV control | | | *lin-39* RNAi | | |
| no sperm | 0 | 0 | 0 | 48 | 34 | 36 |
| no oocyte | 0 | 0 | 0 | 0 | 0 | 0 |
|  |  |  |  |  |  |  |
| **Cochran–Mantel–Haenszel** (**CMH**) **test** | | | | | | |
| EV control (no sperm) vs. *lin-39* RNAi (no sperm) | | | | *p* < 0.0001 | | |

H.

| Exopher: % | | | | | | |
| --- | --- | --- | --- | --- | --- | --- |
|  | EV control | | | *cbd-1* RNAi | | |
| *sem-2(rf)* |  |  |  | 72 | 70 | 68 |
| *sem-2(rf); fem-3(q20)* | 0 | 0 | 0 | 0 | 0 | 0 |
|  |  |  |  |  |  |  |
| **Cochran–Mantel–Haenszel** (**CMH**) **test** | | | | | | |
| *sem-2(rf) vs. sem-2(rf); fem-3(q20)*  *(cbd-1* RNAi) | | | | | *p* < 0.0001 | |

I.

|  | Uterus length: μm | % ALMR with exopher |
| --- | --- | --- |
| WT (day 1) | 295 | 6 |
| WT (day 2) | 389 | 20 |
| *cbd-1(RNAi)* | 214 | 0 |
| SPE-44::AID | 194 | 0 |
| *mex-3(RNAi)* | 377 | 8.7 |
| SPE-44::AID + *sem-2(rf)* | 582 | 86 |
| SPE-44::AID + *lin-39(RNAi)* | 551 | 50 |
| *gad-1(RNAi)* | 370 | 10 |
| *sem-2(rf)* | 520 | 83 |
| *egl-9(*Δ*)* | 498 | 53 |
| egl-3(Δ) | 495 | 83 |
|  |  |  |
| **Correlation** | | |
| Pearson r = 0.8839 | *p* = 0.0003 | |
