# Supplementary material for: Mechanical force of uterine occupation enables large vesicle extrusion from proteostressed maternal neurons
Source: eLife. 2024 Sep 10;13:RP95443. doi: 10.7554/eLife.95443 (PMC11386954; doi:10.7554/eLife.95443)
Supplement: Figure 7—source data 1. [file elife-95443-fig7-data1.docx]

Figure 7-source data: **Numerical data**

A.

| *lin-39(RNAi)* | | | |
| --- | --- | --- | --- |
| Hours after L4 sync | Exopher: % | | |
| 14 |  | 0 | 0 |
| 16 | 0 | 0 | 0 |
| 18 | 0 | 0 | 0 |
| 19 | 0 | 2 | 0 |
| 20 | 12 | 10 | 4 |
| 21 | 24 | 16 | 8 |
| 22 | 34 | 24 | 10 |
| 38 | 48 | 42 | 36 |
| 42 | 50 | 44 | 38 |

D.

| Exopher: % | |
| --- | --- |
| Injected | Control |
| 40 | 0 |
| 22 | 0 |
| 14 | 0 |

|  | Exopher (+) | Exopher (-) |
| --- | --- | --- |
| Control | 0 | 23 |
| Injection | 6 | 17 |
| p-value for Chi-square test | 0.0086 | |

E.

| Exopher: % | |
| --- | --- |
| *lin-39(RNAi)* | EV control |
| 0 | 0 |
| 0 | 0 |
| 7.1 | 0 |
| 17 | 0 |
| 17 | 0 |
| 0 | 0 |

|  | Exopher (+) | Exopher (-) |
| --- | --- | --- |
| Control | 0 | 41 |
| Injection | 3 | 45 |
| p-value for Chi-square test | 0.1034 | |
